# Involvement of CCN1 Protein and TLR2/4 Signaling Pathways in Intestinal Epithelial Cells Response to *Listeria monocytogenes*

**DOI:** 10.3390/ijms23052739

**Published:** 2022-03-01

**Authors:** Cong Zhou, Yafang Zou, Yuanyuan Zhang, Shuang Teng, Keping Ye

**Affiliations:** Jiangsu Collaborative Innovation Center of Meat Production and Processing, Quality and Safety Control, College of Food Science and Technology, Nanjing Agricultural University, Nanjing 210095, China; 2019108059@njau.edu.cn (C.Z.); 2021108056@stu.njau.edu.cn (Y.Z.); 2020808123@stu.njau.edu.cn (Y.Z.); tengshuang@njau.edu.cn (S.T.)

**Keywords:** *Listeria monocytogenes*, intestine epithelial cells, CCN1, TLR2/4 pathways

## Abstract

CCN1 is well studied in terms of its functions in injury repair, cell adhesion survival and apoptosis, bacterial clearance and mediation of inflammation-related pathways, such as the TLR2/4 pathways. However, the role of CCN1 protein and its interaction with TLR2/4 pathways in intestinal epithelial cells was not elucidated after *Listeria monocytogenes* infection. The results of this study confirm that *L. monocytogenes* infection induced intestinal inflammation and increased the protein expression of CCN1, TLR2, TLR4 and p38, which followed a similar tendency in the expression of genes related to the TLR2/4 pathways. In addition, organoids infected by *L. monocytogenes* showed a significant increase in the expression of CCN1 and the activation of TLR2/4 pathways. Furthermore, pre-treatment with CCN1 protein to organoids infected by *L. monocytogenes* could increase the related genes of TLR2/4 pathways and up-regulate the expression of *TNF*, and increase the count of pathogens in organoids, which indicates that the interaction between the CCN1 protein and TLR2/4 signaling pathways in intestinal epithelial cells occurred after *L. monocytogenes* infection. This study will provide a novel insight of the role of CCN1 protein after *L. monocytogenes* infection in the intestine.

## 1. Introduction

*Listeria monocytogenes* is a short Gram-positive flagellar and ubiquitous intracellular bacterium that causes listeriosis in immunocompromised individuals, with a case fatality rate of up to 30% [1]. While *L. monocytogenes* outbreaks are common and generally attributable to highly contaminated foods, most cases of listeriosis are sporadic.

Upon infection, intestinal epithelial cells, which respond to intruders and their cellular molecules by displaying an inflammatory state, play a crucial role in the immune response to pathogens [2]. Toll-like receptors (TLRs), expressed in the intestinal epithelial cells, are the phylogenetically conserved mediators of innate immunity that can discriminate intestinal microbiota and respond to pathogenic microbes by recognizing pathogen-associated molecular patterns (PAMPs) [3,4]. Intestinal epithelial cells, which constitute a single monolayer of cells found at the mucosal surface, express several TLRs, including TLR2, TLR4, TLR5 and TLR9, with the location of these being restricted to either the apical or the basolateral surface, or both [5]. During *L. monocytogenes* infection, TLR2 could recognize peptide glycan and lipoproteins [6], and TLR5 and TLR9 could recognize flagellin and bacterial DNA, respectively [7]. Although TLR4 was commonly reported to act as a signaling receptor of lipopolysaccharide (LPS), a Gram-negative bacterial component, in a recent study, TLR4 of macrophages were activated through cellular communication network factor 1 (*CCN1*), which could recognize peptidoglycan (PGN) of *S. aureus* [8]. Upon recognizing PAMPs, TLR2 or TLR4 could lead signaling to produce various inflammatory cytokines through the activation of myeloid differentiation factor 88 (MyD88)-dependent and -independent pathways [9]. In the MyD88-dependent pathway, TLR2 or TLR4 could activate IL-1 receptor-associated kinase (IRAK), which then proceeded to activate a complex signaling of TGF-β-activated kinase (TAK1). Lastly, the gene expression of regulatory factors of p38 (the MAPKs family) was activated, so as to induce the production of proinflammatory cytokines and chemokines [10].

*CCN1* encodes a 42 kDa matricellular protein that could exhibit diverse and different functions, thereby involving it in distinct and complex processes, such as inflammation and injury repair in the skin, liver and gut [11,12,13]. For instance, a study showed that the CCN1 protein was up-regulated in the intestinal tissue that was subjected to ischemia [14]. In addition, Jun et al. (2020) found that CCN1 protein could activate TLR2 or TLR4 by direct binding to those receptors, leading to MyD88-dependent expression of inflammatory cytokines and chemokines [8], whereas, the role of CCN1 protein and the interaction between the CCN1 protein and TLR2/4 pathways in regulating innate immune responses of intestinal epithelial cells during *L. monocytogenes* infection is largely unknown. 

Investigations of pattern recognition receptors (PRRs) were mostly focused on the immune cell’s response to enteric pathogens [15,16,17]; however, PRRs of the intestinal epithelium also played an important role in innate immunity after bacterial infection [18]. As for the cell models in vitro, compared with other traditional cell models, intestinal organoids were cell spheroids that could differentiate into the various intestinal epithelial cell types, which could express several TLRs, including TLR2, TLR4, TLR5 and TLR9 [5]. The model allowed the investigation of intestinal function and direct interactions with microbes. In addition, researchers had used intestinal organoids to model the natural infection of *L. monocytogenes* [19], *Salmonella* [20] and pathogenic *Escherichia coli* strains [21,22], which provided important insights into pathogenesis in the intestine.

Therefore, this study, based on mice and intestinal organoids as in vivo and in vitro models, respectively, aimed to explore the changes in the CCN1 protein and TLR2/4 signaling pathways of intestinal epithelial cells, as well as their interaction after *L. monocytogenes* infection, which were expected to provide a novel insight on the role of CCN1 protein after *L. monocytogenes* infection in the intestine.

## 2. Results

### 2.1. The Pathological Changes in Mice after L. monocytogenes Infection

**Figure 1:**
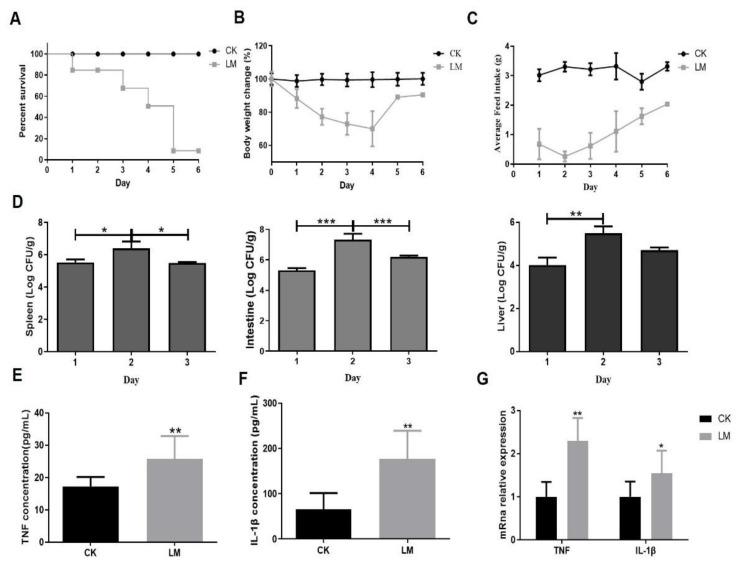
The pathological changes in mice after *L. monocytogenes* infection. (**A**) Time course of survival rate of mice. (**B**) Time course of body weight change in mice. (**C**) Time course of average feed intake of mice. (**D**) *L. monocytogenes* burden in the spleen, intestine and liver. (**E**,**F**) The concentration of TNF and IL-1β in jejunum homogenate of mice. (**G**) mRNA levels of *TNF* and *IL-1β* from homogenized jejunum samples. * *p* < 0.05, ** *p* < 0.01, *** *p* < 0.001.

The pathological changes were analyzed in mice gavaged with either PBS (CK) or *L. monocytogenes* (LM). Compared with the CK group (mice or organoids without infection, CK group), mice infected with *L. monocytogenes* by oral gavage died starting from day one, and the survival rate dropped sharply at 5 days (Figure 1A). Moreover, the body weight and feed intake of mice were much lower than the control group after infection (Figure 1B,C). The colonization of *L. monocytogenes* in the spleen, intestine and liver reached a peak on day two (Figure 1D). Figure 1E,F show that the concentrations of IL-1β and TNF in jejunum homogenate were significantly higher than that in the CK group, which was in agreement with the results of mRNA expression levels of IL-1β and TNF in the jejunum (Figure 1G). These results confirm that infection of this *L. monocytogenes* strain could lead to serious pathological changes in mice.

### 2.2. The Changes in CCN1 Protein and TLR2/4 Signaling Pathways Response to L. monocytogenes Infection in Mice

**Figure 2:**
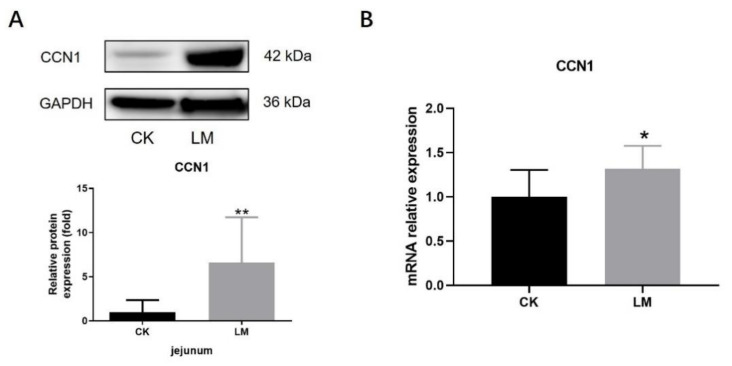
The changes in CCN1 response to *L. monocytogenes* infection in mice. (**A**) Western blot of CCN1 in the jejunum. (**B**) mRNA levels of *CCN1* in the jejunum. * *p* < 0.05, ** *p* < 0.01.

**Figure 3:**
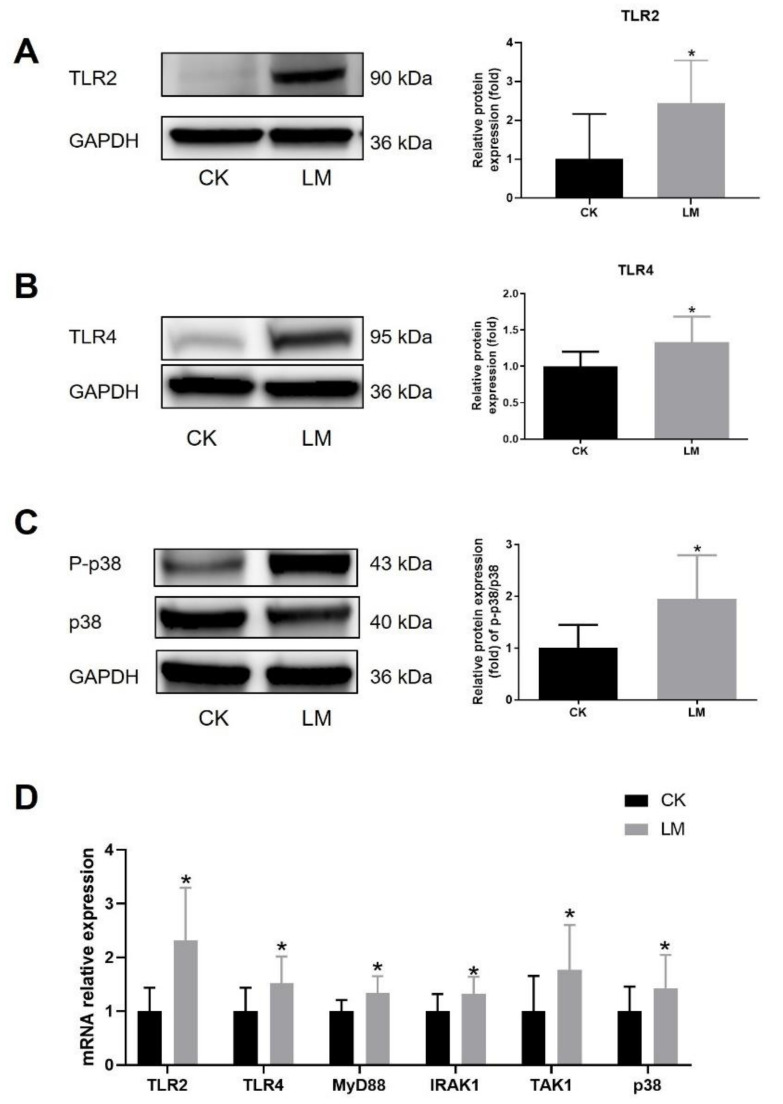
The increase in components in TLR2/4 signaling pathways upon *L. monocytogenes* infection in mice. (**A**–**C**) Western blot of TLR2, TLR4 and P-p38/p38 in the jejunum. (**D**) mRNA levels of *TLR2*, *TLR4*, *MyD88*, *IRAK1*, *TAK1* and *p38* from homogenized jejunum samples. * *p* < 0.05.

In order to determine the changes in CCN1 protein and TLR2/4 signaling pathways, the jejuna of mice, gavaged with either PBS (CK) or *L. monocytogenes* (LM), were collected. After *L. monocytogenes* infection, the protein expression of CCN1 had a 6.63-fold increase (*p* < 0.05) (Figure 2A), and the mRNA relative expression of *CCN1* also increased significantly (Figure 2B), which indicated that *L. monocytogenes* infection stimulated the expression increase in CCN1 in the jejuna of mice. Under this circumstance, Western blot analysis showed that the protein expression of TLR2 and TLR4, which could be activated through CCN1 protein in the macrophages, increased significantly in the LM group (Figure 3A,B), and the protein expression of P-p38/p38 that was downstream of the TLR2/4 signaling pathways increased 1.94-fold in the LM group (Figure 3C). In the meantime, the mRNA expression levels of genes related to the TLR2/4 signaling pathway (*TLR2*, *TLR4*, *MyD88*, *IRAK1*, *TAK1* and *p38*) were significantly increased after *L. monocytogenes* infection (Figure 3D). These results indicate that *L. monocytogenes* infection increased the expression of CCN1, which followed a similar tendency in the expression of the TLR2/4 signaling pathways.

### 2.3. The Damage of Small Intestinal Organoids after L. monocytogenes Infection

**Figure 4:**
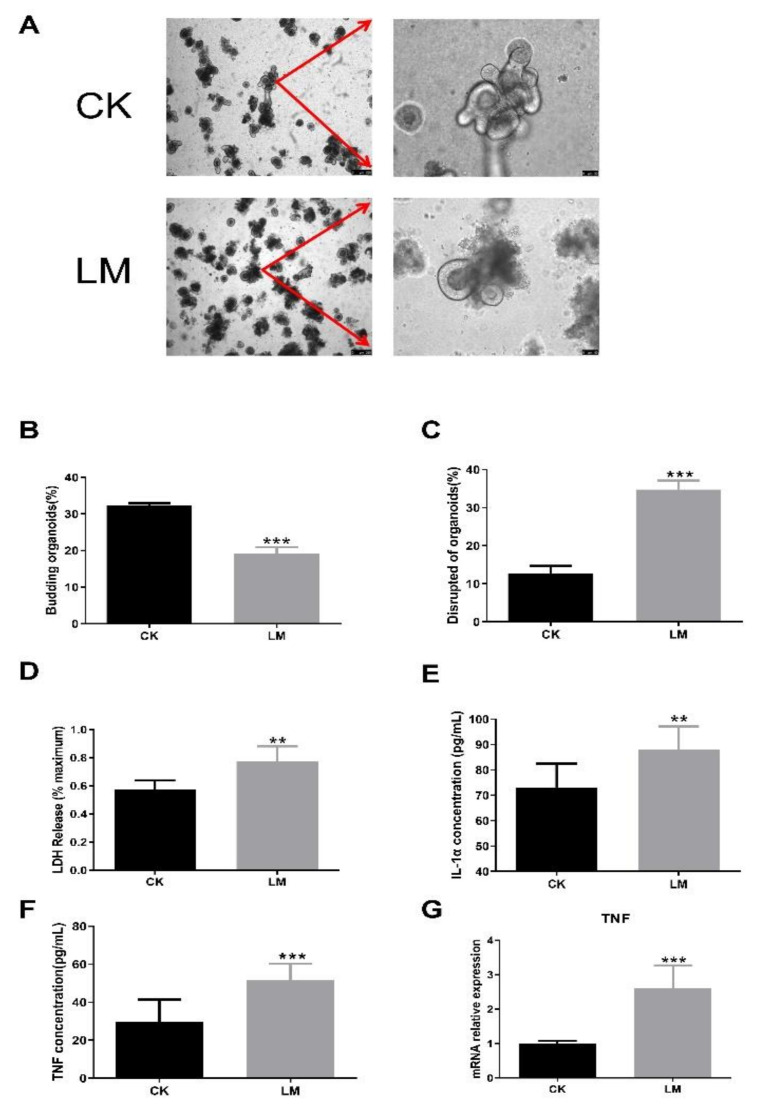
The damage of small intestinal organoids after *L. monocytogenes* infection. (**A**) The morphological changes in organoids after *L. monocytogenes* infection. (**B**,**C**) The budding percentage, and mortality, of organoids after *L. monocytogenes* infection. (**D**) LDH release in the supernatant. (**E**,**F**) The concentration of TNF and IL-1β in the supernatant. (**G**) mRNA levels of *TNF* from organoids samples. ** *p* < 0.01, *** *p* < 0.001.

Intestinal organoids, treated with culture medium (CK) or *L. monocytogenes* (LM), were used to verify the effect of *L. monocytogenes* infection on the intestinal epithelial cells, which was also an important line against infection except for immune cells in the intestine. Figure 4A shows that organoids infected by *L. monocytogenes* were partially lysed at 18 h compared with the normal budding in the control group, and the budding rate of organoids was significantly decreased, and the mortality of organoids was significantly increased, in the LM group (Figure 4B,C). Moreover, LDH (lactate dehydrogenase) release in the supernatant was increased significantly in the LM group, which indicated the damage to the cell membrane of small intestinal organoids (Figure 4D). As shown in Figure 4E,F, *L. monocytogenes* infection induced a significant increase in the protein expression levels of TNF and IL-1β at 18 h of co-culture, and the mRNA expression levels of *TNF* followed a similar significant increase to the protein expression (Figure 4G). These results reflect that *L. monocytogenes* infection had significant damage to the organoids.

### 2.4. The Changes in CCN1 Protein and TLR2/4 Signaling Pathways Response to L. monocytogenes Infection in Organoids

**Figure 5:**
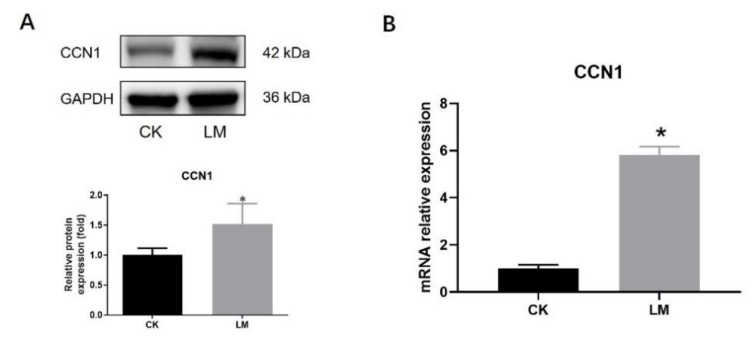
The changes in CCN1 protein response to *L. monocytogenes* infection in organoids. (**A**) Western blot of CCN1 in organoids. (**B**) mRNA levels of *CCN1* from organoids samples. * *p* < 0.05.

**Figure 6:**
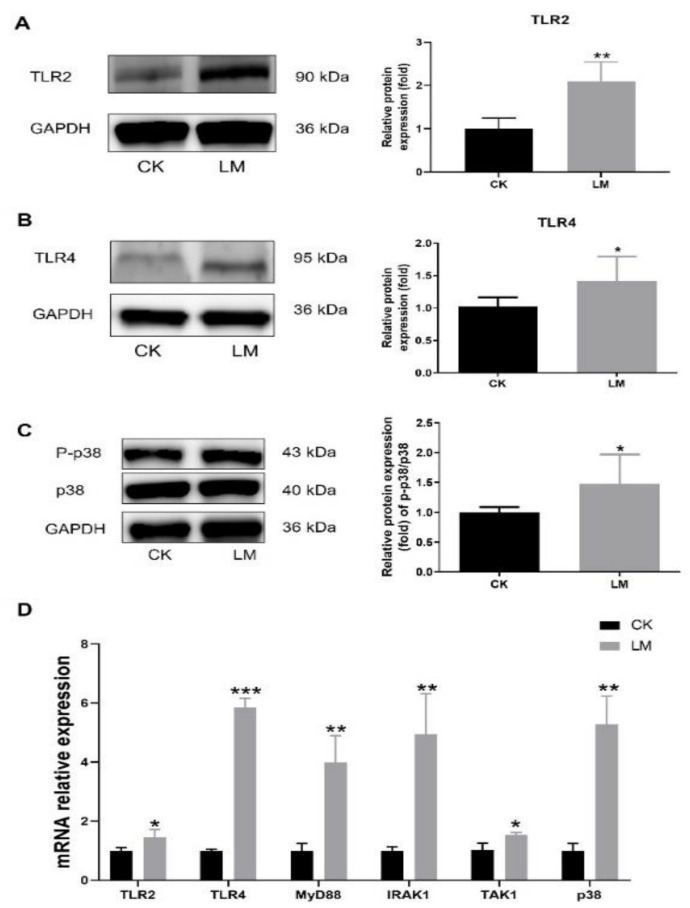
The increase in components in TLR2/4 signaling pathways upon *L. monocytogenes* infection in organoids. (**A**–**C**) Western blot of TLR2, TLR4 and P-p38/p38 in organoids. (**D**) mRNA levels of *TLR2*, *TLR4*, *MyD88*, *IRAK1*, *TAK1* and *p38* from organoids samples. * *p* < 0.05, ** *p* < 0.01, *** *p* < 0.001.

Intestinal organoids were used to further confirm the changes in the CCN1 protein and TLR2/4 signaling pathways of intestinal epithelial cells in response to *L. monocytogenes* infection. Obviously, the protein and mRNA expression levels of CCN1 were increased significantly in the LM group (Figure 5A,B). Furthermore, compared with the control group, the protein expression of TLR2, TLR4 and P-p38/p38 also showed a significant increase (Figure 6A–C), and the mRNA expression levels of TLR2/4-pathway-related genes (*TLR2*, *TLR4*, *MyD88*, *IRAK1*, *TAK1*, and *p38*) increased significantly (Figure 6D). Overall, the results of organoids confirmed those of the mice findings, which indicated that the expression of CCN1 was increased and the TLR2/4 signaling pathway was activated after *L. monocytogenes* infection in the intestinal epithelial cells.

### 2.5. The Changes in TLR2/4 Signaling Pathways Response to L. monocytogenes Infection in Organoids Pre-Treated with CCN1 Protein

**Figure 7:**
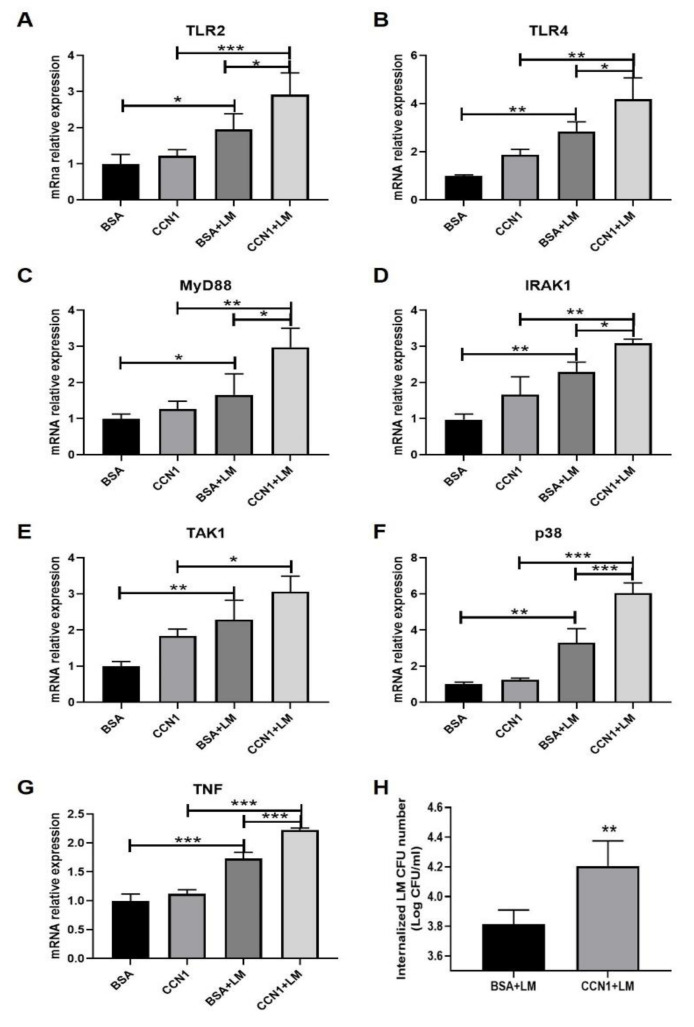
The changes in TLR2/4 signaling pathways in response to *L. monocytogenes* infection in organoids pre-treated with CCN1 protein. (**A**–**G**) mRNA levels of *TLR2*, *TLR4*, *MyD88*, *IRAK1*, *TAK1* and *p38* from organoids samples. (**H**) The number of *L. monocytogenes* in the organoids. * *p* < 0.05, ** *p* < 0.01, *** *p* < 0.001.

The results show that the pre-treatment of CCN1 protein significantly increased the mRNA expression levels of TLR2/4-pathway-related genes (*TLR2*, *TLR4*, *MyD88*, *IRAK1*, *TAK1* and *p38*) (Figure 7A–F), which could lead to the increase in the mRNA expression level of *TNF* after *L. monocytogenes* infection (Figure 7G). Furthermore, it was found that the pre-treatment of CCN1 protein could increase the counts of *L. monocytogenes* in intestinal organoids (Figure 7H). These results illustrate that pre-treatment of CCN1 protein could increase the expression of the TLR2/4 signaling pathways of intestinal epithelial cells and the susceptibility of intestinal organoids to *L. monocytogenes* infection.

## 3. Discussion

The innate immunity of intestinal epithelial cells plays an important part in defending against many enteric bacterial pathogens [23]. The results of this study show a significant up-regulated response in TNF and IL-1β expression levels caused by *L. monocytogenes* infection in mice and organoids, which indicates that *L. monocytogenes* caused the inflammation of intestinal epithelial cells. This corroborated the studies of He et al., suggesting that *L. monocytogenes* infection could cause the increased production of TNF and IL-1β in intestinal cells [24]. 

CCN1 protein has been found to exhibit diverse functions, including cell adhesion survival and apoptosis, and plays an important role in inflammation and tissue repair [8,25,26,27]. In this study, it was first found that *L. monocytogenes* infection could increase the mRNA and protein expression of CCN1 in mice and intestinal organoids. However, as for other pathogen infections, it was found that *S. aureus* and its supernatants could induce the expression of CCN1 in epithelial cells [28], while the expression of CCN1 was inhibited by *Salmonella enterica* [29]. Furthermore, a recent reference reported that CCN1 of macrophages, functioning as a pattern recognition receptor (PRR), opsonized Gram-positive and -negative bacteria through binding peptide glycan (PGN) and lipopolysaccharide (LPS), respectively [8]. Therefore, after *L. monocytogenes* infection in vivo and in vitro, the PGN of bacteria and the damage of intestines may be the reasons for the increase in CCN1 expression in intestinal epithelial cells.

The stimulation of TLRs activates at least two major downstream signaling pathways, nuclear factor (NF)-κB and mitogen-activated protein kinases (MAPKs), to induce inflammation after pathogen infection [30]. This study showed that *L. monocytogenes* infection caused the increase in TLR2 expression in mice and organoids, which was consistent with the previous studies [15,31,32]. In addition, to our surprise, it was found that the mRNA and protein expression levels of TLR4, in vivo and in vitro, were increased after *L. monocytogenes* infection. In the literature, it was generally acknowledged that TLR4 did not recognize any components of *L. monocytogenes* based on the data generated from TLR4-deficient mice and in vitro activation assays using TLR4 transfected human embryonic kidney (HEK) 293 cells [33,34]. However, a study demonstrated that the TLR4 signaling pathway was activated in Pregnane X Receptor (PXR) deficient mice after *L. monocytogenes* infection, and the inflammation induced by TLR4 signaling was detrimental to host defense against the infection [35]. Our study found that the related genes of the MyD88-dependent signaling pathway (*MyD88*, *IRKA1* and *TAK1*), which could be activated by TLR2 or TLR4, were increased significantly in intestinal epithelial cells infected by *L. monocytogenes*. This phenomenon was consistent with the previous report of *L. monocytogenes* infection [36,37]. In addition, the ratio of P-p38/p38 in protein level and the *p38* in mRNA level was also up-regulated in mice and intestinal organoids, which indicates that the downstream MAPK pathway had been activated to induce inflammation after *L. monocytogenes* infection [38].

CCN1 can, by itself, induce inflammation through physical interaction with TLR2 or TLR4 to activate MyD88-dependent signaling in mice and macrophages [8]. Based on the results of the increase in the expression of CCN1 and the activation of TLR2/4 signaling pathways in vivo and in vitro, we speculated that the interaction between CCN1 protein and TLR2/4 signaling pathways occurred after *L. monocytogenes* infection. To verify this hypothesis, intestinal organoids were pre-treated with or without CCN1 protein prior to infection, and the results show that pre-treatment with CCN1 protein increased the expression of the related genes in the TLR2/4 signaling pathways, which followed a similar pattern to the mRNA expression level of *TNF* after infection. Moreover, the pre-treatment of CCN1 protein could increase the counts of *L. monocytogenes* in intestinal organoids.

**Figure 8:**
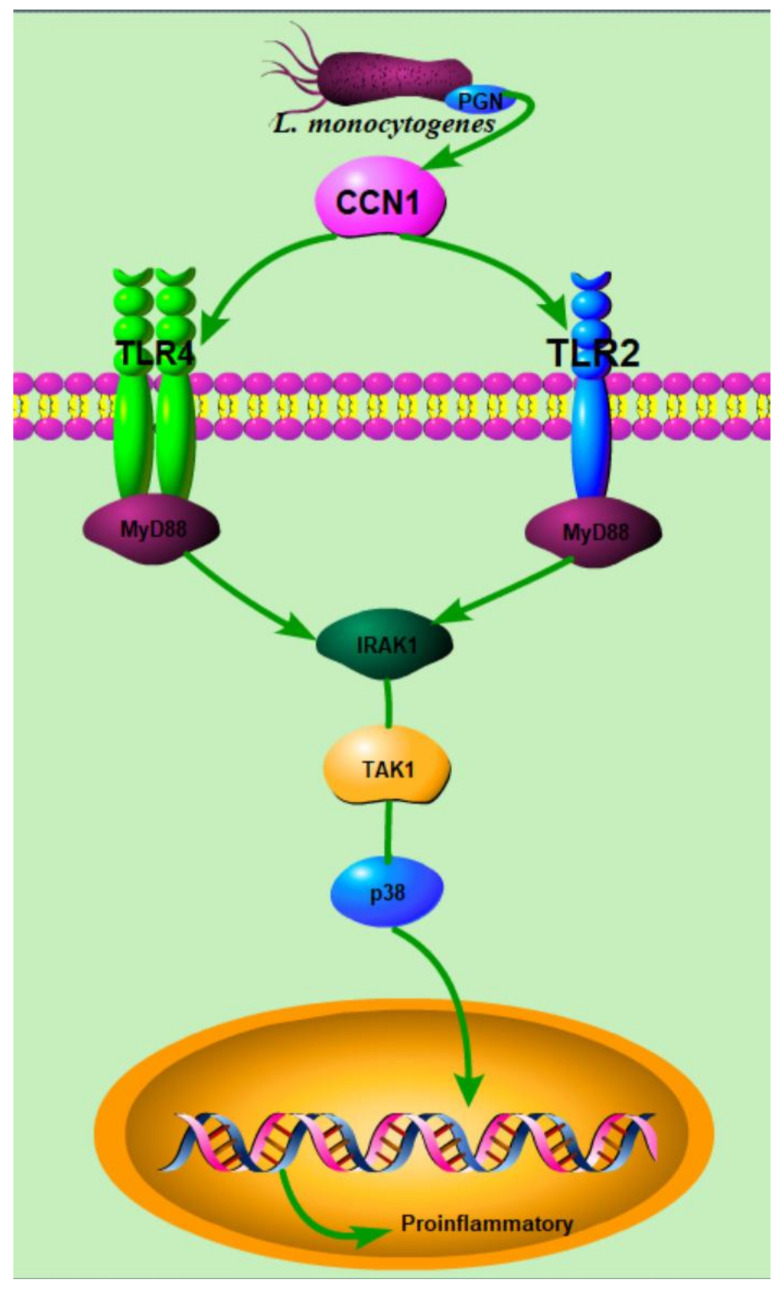
The conjectured mechanism of the intestinal epithelial cell’s response to *L. monocytogenes* infection.

In summary, this study, using mice and intestinal organoids, indicates that *L. monocytogenes* infection caused the pathological changes in mice and the disruption of organoids, and induced the inflammation of the intestinal epithelial cells. Furthermore, *L. monocytogenes* infection caused the increased expression of CCN1 and the activation of TLR2/4 signaling pathways, and the interaction between these occurred after infection, which may be the reason for the increase in pro-inflammatory cytokines secretion (Figure 8). Moreover, the pre-treatment of CCN1 protein could increase the susceptibility of intestinal organoids to *L. monocytogenes*. Further studies can be focused on CCN1-deficient mice to confirm the role of CCN1 and the specific mode of interaction between CCN1 and TLR2/4 signaling pathways after infection, which may prompt novel therapies for pathogen infections. 

## 4. Materials and Methods

### 4.1. Bacterial Strain Culture

The challenge organism for this experiment was *L. monocytogenes* 10403s. It was grown with agitation overnight at 37 °C in Brain Heart Infusion (BHI) broth, and supplemented with 5 μg mL^−1^ erythromycin.

### 4.2. Animals and Intestinal Organoids 

#### 4.2.1. Ethics Statement

This study was carried out in accordance with the National Institutes of Health guidelines for the performance of animal experiments. All operations related to animal experiments were examined and approved by the Nanjing Agriculture University Committee on Animal Resources Committee (Approval ID: NJAU.No20210305009).

#### 4.2.2. Animals

Four-week-old C57BL/6 mice (specific-pathogen-free (SPF) female) were purchased from the Animal Research Centre of Yang Zhou University (production license number: SCXK(SU)2017-0007). Three independent animal experiments were carried out in this study. For the first animal experiment, 22 mice were randomly divided into two groups, which were orally administered sterile PBS (200 μL, control group, CK, n = 10) and *L. monocytogenes* 10403s (200 μL, 10^9^ CFU/mL, LM, n = 12). Then, the survival, average feed intake and average body weight of mice were recorded every day. For the second animal experiment, 15 mice were orally administered *L. monocytogenes* 10403s (200 μL, 10^9^ CFU/mL) for determining the number of bacteria in the spleen, liver and jejunum after 24, 48, and 72 h of infection (3 survivors were used in each time point). For the third animal experiment, mice were randomly divided into two groups, which were orally administrated sterile PBS (200 μL, control group, CK, n = 10) and *L. monocytogenes* 10403s (200 μL, 10^9^ CFU/mL, LM, n = 20). They were sacrificed on day 4, and jejunum tissue samples and contents were collected for further analysis. 

#### 4.2.3. Intestinal Organoids

##### Isolation and Culture of Intestinal Organoids

The small intestine of 4-week-old C57BL/6 wild-type mice was cut into small pieces and washed several times with cold PBS. The pieces were then incubated with Gentle Cell Dissociation Reagent (Stem Cell, Vancouver, Canada) for 15 min at 20 °C, and crypts were detached from basal membrane by vigorous shaking. After incubation, crypts enriched in the supernatant were passed through a 70 μm strainer and centrifuged at 300× *g* for 5 min at 4 °C. The cells were counted, and then, about 200 crypts in each well were resuspended by 25 μL Matrigel (Corning, NY, USA) and 25 μL IntestiCult^TM^ OGM Mouse Basal Medium (Stem cell, Vancouver, Canada), and then plated in 24-well plates. After polymerizing at 37 °C for 20 min, the culture medium supplemented with penicillin–streptomycin (100 U/mL) was added into the gel in each well. The medium was changed every 2–3 days.

##### *L. monocytogenes* Infection of Organoid Cells

The methods of organoid infection were referenced by Huang et al. [19], with some modifications. Briefly, after removing the Matrigel with cold PBS, organoids of each well were exposed to the culture medium with *L. monocytogenes* (100 μL, 10^8^ CFU/mL) for 1 h. Subsequently, the organoids were reseeded with Matrigel and cultured with medium containing gentamicin (100 μg/mL, Gbico) for 18 h. Finally, in order to evaluate infection damage, total organoid numbers, budding organoids and dead organoid numbers per well were counted under a light microscope (Leica, Wetzlar, Germany).

To verify the interaction between CCN1 protein and TLR2/4 signaling pathways, organoids of each well were pre-treated with 100 μL CCN1 protein (4 μg/mL, CUSABIO) for 1 h (marked as CCN1), and organoids of each well were pre-treated with 100 μL BSA (4 μg/mL) as the control group (marked as BSA). Then, the intestinal organoids of each well were infected by *L. monocytogenes* (100 μL, 10^8^ CFU/mL) for 1 h (BSA + LM: organoids pre-treated with BSA for 1 h prior to infection; CCN1 + LM: organoids pre-treated with CCN1 protein for 1 h prior to infection). Subsequently, the organoids were reseeded with Matrigel and cultured with medium containing gentamicin (100 μg/mL, Gbico) for 18 h.

### 4.3. The Number of L. monocytogenes in Target Organs and Organoids

After 24, 48 and 72 h of infection, the jejuna, spleens and livers were weighed and grounded with a 200-mesh sieve, respectively. Then, the tissue homogenate was resuspended with 1 mL sterile water for 30 min at room temperature. After performing 1:10 serial dilutions, 1 mL suspension of each dilution was inoculated onto BHI agar plates supplemented with 5 μg/mL erythromycin and incubated for 48 h at 37 °C to determine the count of the *L. monocytogenes* (Log CFU/g).

To assess the total number of *L. monocytogenes* infecting organoids with or without CCN1 pre-treatment, the organoids were resuspended with 1 mL sterile water for 30 min at room temperature, and the serial dilution and incubation of *L. monocytogenes* was described above. After incubation, the CFU/mL was counted.

### 4.4. ELISA

After *L. monocytogenes* infection, the production of TNF and IL-1β in jejunum contents and organoid culture supernatants were analyzed using Mouse TNF ELISA kits (NEBIOSCIENC, China) and Mouse IL-1β ELISA kits (NEBIOSCIENC, China) according to the manufacturer’s protocols, respectively.

### 4.5. LDH Release Assay 

After the organoids were co-cultured with *L. monocytogenes* for 18 h, the LDH in cell culture supernatants was collected and detected by LDH Cytotoxicity Assay Kit according to the manufacturer’s protocols.

### 4.6. Real-Time Quantitative PCR

Total RNA from the jejunum and organoid samples was extracted with TRIzol (Ambion, Austin, TX, USA) and quantified by spectrophotometry (NanoDrop ND1000, USA), after which reverse transcription PCR was performed. Then, 1 μL of template cDNA was reacted with a master mix in a final volume of 10 μL. The qPCR thermal cycling conditions were as follows: 30 s at 95 °C, followed by 40 cycles of 10 s at 95 °C and 30 s at 60 °C. Primers for specific genes are listed in Table 1.

### 4.7. Western Blot

Tissue samples and organoids were lysed in RIPA buffer containing a protease and phosphatase inhibitor cocktail. Protein concentrations of the lysed samples were detected using a bicinchoninic acid (BCA) assay kit (Thermo Scientific, Waltham, MA, USA), after which samples containing 5× load buffer were heated at 95 °C for 5 min. Equal amounts of protein in various samples were separated by 4–20% SDS-PAGE and transferred to PVDF membranes (BIO-RAD, Hercules, CA, USA). After that, the membranes were blocked with 5% non-fat milk in TBS with 0.1% Tween-20 for 1 h and then incubated with rabbit anti-GAPDH (abcam, Cambridge, UK, RRID: AB_2107448), rabbit anti-CCN1(1:1000, Affinity, Cincinnati, OH, USA, RRID: AB_2838216), rabbit anti-TLR2 (1:1000, Affinity, Cincinnati, OH, USA, RRID: AB_2838958), rabbit anti-TLR4 (1:1000, abcam, Cambridge, UK, RRID: AB_300696), rabbit anti-p38 (1:1000, CST, Beverly, MA, USA, RRID: AB_10999090) and rabbit p-p38 (1:1000, CST, Beverly, MA, USA, RRID: AB_331641) overnight. After washing, goat anti-rabbit secondary antibodies (Bioss, 1:1000) were used to incubate the membranes for 2 h. Finally, the optical protein bands were developed using the efficient chemiluminescence (ECL) kit, and light emission was captured using the Versa DOC 4000 imaging system.

### 4.8. Statistical Analysis

The results are expressed as the means ± SD. A *t*-test was employed to determine the significant differences between two groups, and one-way ANOVA was employed to determine the significant differences among multiple groups. The significance levels are shown as * *p* < 0.05, ** *p* < 0.01 and *** *p* < 0.001. Data were combined from at least three independent experiments unless otherwise stated.

## Figures and Tables

**Table 1:** Primer sequences used for RT-qPCR.

Target Genes	Primer Sense (5′-3′)	Primer Antisense (5′-3′)
*CCN1*	CTGCGCTAAACAACTCAACGA	GCAGATCCCTTTCAGAGCGG
*TLR2*	GGACATCCCCTTCCCTCACTTC	ACGGGCAGTGGTGAAAACT
*TLR4*	TTCAGAGCCGTTGGTGTATC	CCCATTCCAGGTAGGTGTTT
*MyD88*	CCTGCGGTTCATCACTAT	GGCTCCGCATCAGTCT
*IRAK1*	CCACCCTGGGTTATGTGCC	GAGGATGTGAACGAGGTCAGC
*TAK1*	ATGTTTGTCGTGCCTTTCTCT	AAGGGTTTCCGGCGTGTTAT
*p38*	CAGAAACTGACGGACGACCA	CAGCTCGGCCATAATGCAAC
*TNF*	TACTGAACTTCGGGGTGATTGGTCC	CAGCCTTGTCCCTTGAAGAGAACC
*IL-1β*	GCTTGGTGATGTCTGGTCCA	AACACGCAGGACAGGTACAG
*GAPDH*	ATGGTGAAGGTCGGTGTGAA	TGGAAGATGGTGATGGGCTT

## Data Availability

Not applicable.
